# Microcapsulated biocides for the targeted control of invasive bivalves

**DOI:** 10.1038/s41598-019-55392-4

**Published:** 2019-12-11

**Authors:** Feng Tang, David C. Aldridge

**Affiliations:** 1 Department of Zoology, University of Cambridge, The David Attenborough Building, Pembroke Street, Cambridge, CB2 3QZ United Kingdom; 2 BioRISC, St. Catharine’s College, Cambridge, CB2 1RL UK

**Keywords:** Invasive species, Invasive species

## Abstract

Invasive alien species (IAS) are one of the greatest drivers of ecological change. Typically, control uses chemical agents that often are ineffective, harmful to non-target organisms, and environmentally persistent. Bivalves are frequently high impact IAS, but have proven particularly hard to control due to their valve-closing response when exposed to conventional control agents. Microencapsulation of biocides with edible coatings represents a highly targeted delivery route, bypassing avoidance responses and accumulating in bivalves through their prodigious filter feeding. Uneaten microcapsules degrade and become biologically inactive within hours thus reducing potential impacts on non-target biota. We manufactured two new formulations of microcapsules (BioBullets). Particles were designed to mimic natural food particles (algae) in terms of size (9.5 ± 0.5 to 19.4 ± 1.3 SE µm diameter), buoyancy (near neutral) and shape (spherical). Laboratory exposures demonstrated that two formulations effectively controlled the Gulf wedge clam *Rangia cuneata*, an IAS currently spreading rapidly through Europe. A single dose of 2–6 mg L^−1^ of the active ingredient in a static system achieved 90% mortality after 30 days of exposure. Microencapsulation offers an effective and targeted management tool for rapid responses following the early detection of both Gulf wedge clams and many other filter-feeding IAS, and may be especially effective in closed systems or where populations remain very localised.

## Introduction

Aquatic invasive alien species (IAS) threaten native species and biodiversity^1,2^, drive ecosystem change^3–5^, and cause significant economic impacts to industries such as aquaculture, fishing, and tourism^6–9^. Given the widely recognized ecological and economic impacts of aquatic invaders, it is unsurprising that considerable effort has been placed on developing effective management programmes. The new EU legislation (EU Regulation no. 1143/2014) adheres to the three hierarchical measures: (1) prevention, (2) early detection and rapid eradication, and (3) management to combat the invaders^10^. Prevention, which has concentrated on managing international shipping and ballast water^11^, is universally agreed as the cornerstone of managing aquatic invasive species^12^. However, with the numerous vectors that introduction of aquatic species involves^11^, comprehensive prevention of invasion is virtually impossible, even in nations with the most advanced biosecurity protocols^13^. Indeed, the global rate of establishment of non-native species has been unprecedentedly high during recent decades^14,15^, and there is no sign of saturation^16^. When prevention fails, the second tier of management for a species recognised as a high risk^17^ typically involves a rapid response. In situations where a high-risk invader has been detected early, is locally distributed, and there is a low likelihood of reinvasion, then containment or eradication may be considered.

Eradications of aquatic invasive species in open areas have been attempted and sometimes have been successful^18,19^. In over 90% of cases control of marine invasive animals has been mechanical, which involves physical removal of the organism from its introduced range^18^. However, mechanical control usually requires a tremendous input of labour, especially when the established population is large and widespread^18^. Chemical control, on the other hand, is in general a much more cost-effective option with higher success compared with mechanical control^19^. Toxicants applied to aquatic systems will typically diffuse throughout the system and affect the individuals that are otherwise difficult to detect^19^. This characteristic, however, also threatens non-target organisms, and the damage made can be more detrimental than the invasion itself^20,21^. For example, the most common used chemical control agent, chlorine, is a non-specific poison and kills most aquatic organisms^22^.

Chemical control of invasive bivalve molluscs can be especially challenging because they can detect toxins in water and close their shells, thus isolating themselves from the toxins for a prolonged period^23^. Considering the exceptional detrimental ecological and economic impacts caused by invasive bivalves in various ecosystems^24^ and the increasing rate of discovery of non-native bivalves^25^, there is a pressing need to develop effective and targeted control strategies for these taxa.

The recent development of the low-cost and environmentally friendly microencapsulated BioBullet provides a novel approach to the control of IAS bivalves^26–29^. Microencapsulation of control agents with edible, attractive coatings bypasses the shell-closing response seen in most bivalves and results in the immediate and rapid uptake of the active ingredient. BioBullets have been demonstrated to be effective in the control high risk invasive bivalves, such as zebra mussels (*Dreissena polymorpha*)^26,27^ and golden mussels (*Limnoperna fortunei*)^29^. Interestingly, when some native species, such as unionid mussels are exposed to BioBullets, they recognise the products as non-food and reject them upon pseudofaecal threads thus avoiding uptake^26^. This demonstrates that some bivalves are more susceptible than others to BioBullets and that factors including particle size shape and surface texture may be important in affecting ingestion. BioBullets that are uneaten by bivalves degrade within hours to harmless concentrations, thus minimising impacts on the wider biota.

One of the major challenges in manufacturing a microencapsulated biocide is to match the particle size to the feeding preference of the target organism^30^. Filter feeding is not merely a sieving process as consumers capture and retain particles preferentially within certain size ranges^30^. The particle size of previous formulations of BioBullets (101 to 105 μm;^26,27^) was much larger than the preferred prey size range (5–32 μm) of their targeted species, the zebra mussel^31^. Maximising uptake and thus minimising effective doses therefore rests on a closer size matching between microcapsules and diet. The manufacturing of microencapsulated biocides has been advanced substantially in recent years with two new formulations of BioBullets (SB1000 and SB2000) approved for use in UK drinking waters (Regulation 31) by the Drinking Water Inspectorate. Both have also been approved for release into open waters by the UK Environment Agency. In this study, we manufactured and analysed the physical characteristics of the two new formulations of BioBullets that were encapsulated using the advanced technology. We then tested the effectiveness of these two formulations of BioBullets against an IAS currently invading Europe: the Gulf wedge clam, *Rangia cuneata*^32–38^. The use of BioBullets as a potential control agent for *R. cuneata* in Great Britain has been advocated in the national Rapid Risk Assessment commissioned by the GB Non-Native Species Secretariat^39^.

## Results

### Physical characteristics

**Figure 1:**
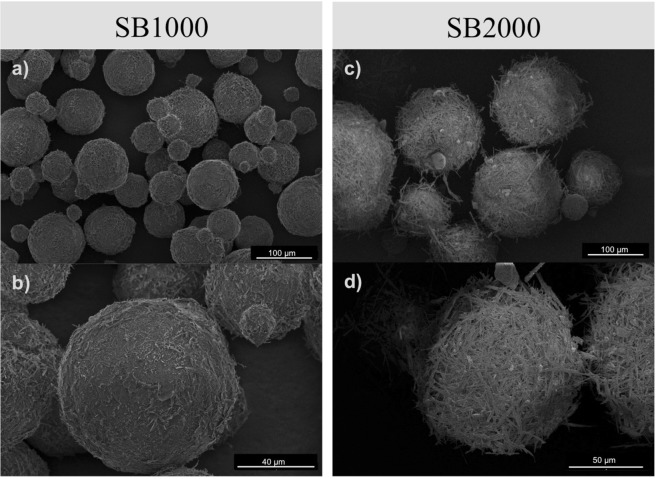
Scanning electron micrographs showing the external appearance of the particles of BioBullets product SB 1000 (**a,b**) and SB 2000 (**c,d**).

The scanning electron micrographs illustrated the external appearance of the microstructure of both of the formulations (Fig. 1). The particles of both of the formulation were spherical. However, the surface structure of the two formulations differed remarkably. While SB1000 particles have a flaky surface (Fig. 1b), SB2000 particles were covered by rod-like structures (Fig. 1d).

**Figure 2:**
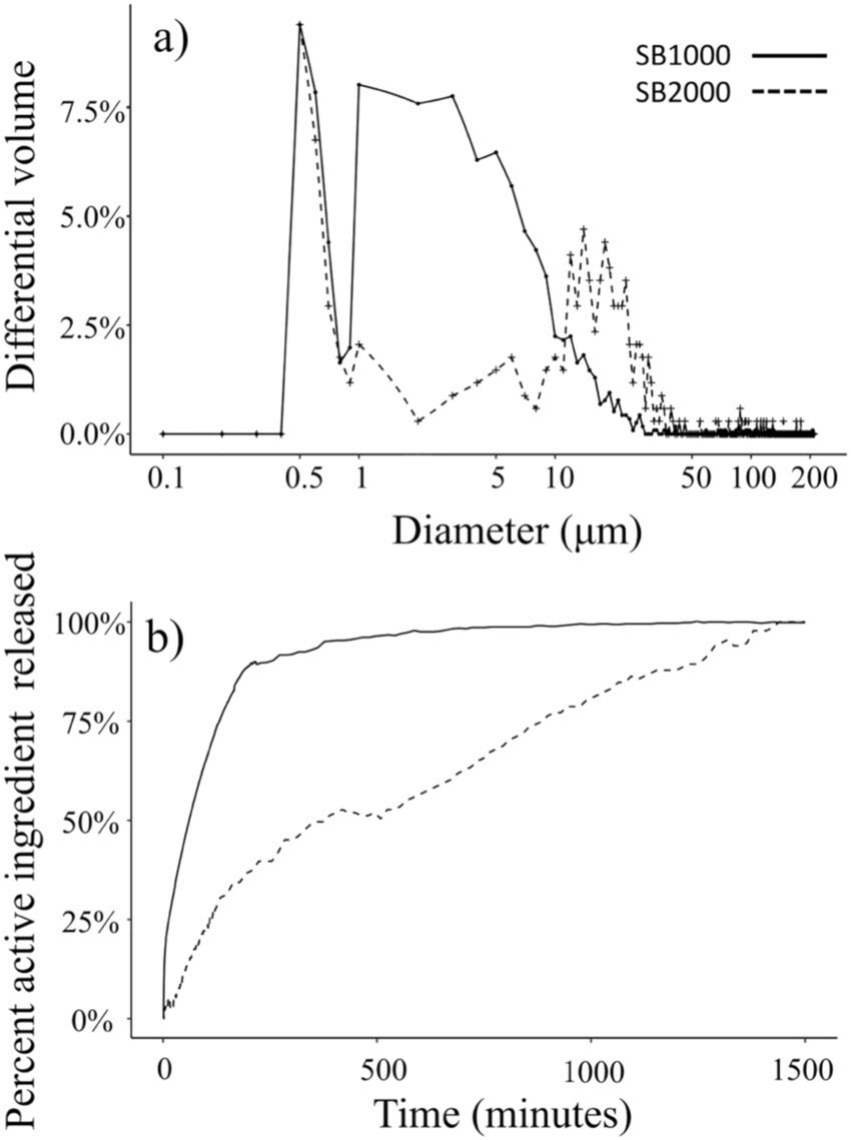
The size distribution (**a**) and release profile (**b**) of the particles in BioBullets product SB1000 and SB2000.

The particles in both formulations followed a bimodal size distribution and had a similar mode between 0.5–1 µm (Fig. 2a). This common mode comprised the coating slurry that was not properly attached to the biocide core. The second modes of each of the size distribution likely showed the actual size distribution of the coated biocide (Fig. 2a). The mean diameter of SB1000 (by volume) was 7.25 ± 0.38 µm (mean ± SE, n = 1161) with the small coating debris included and was 9.47 ± 0.49 µm (mean ± SE, n = 868) when the coating debris was excluded. The particles of formulation SB2000 was much larger compared to the SB1000 (Fig. 1a,c), with a mean diameter of 19.39 ± 1.32 µm (mean ± SE, n = 340) when the coating debris was included, and was 24.69 ± 1.55 µm (mean ± SE, n = 265), when the coating debris was excluded.

In the first 200 minutes, 90% of the active ingredient in SB1000 had been released in water (Fig. 2b). In contrast, the active ingredient in SB2000 released steadily at a much slower rate (Fig. 2b). During the first 6 hours, only 50% of the active ingredient from SB2000 was released into the water. The remaining half of the active ingredient was released at an even slower rate. After 20 hours, 90% of the SB2000 active ingredient was released in water (Fig. 2b). It should be noted that the release profile was produced under standard conditions, which were different from the conditions that we used in the molluscicidal performance experiment. As we used conductivity to measure breakdown in our standard curves it was necessary to use deionised water. However, we would not expect the low ion concentration in the river water used in our experiments to unduly affect release profiles. While release rates of encapsulated biocides usually increase with water temperature^40^, the effect of the temperature used in the standard curve (25 °C) compared with that used in the experimental conditions (10 °C) would likely accelerate breakdown rates by no more than 30 minutes (D. Aldridge, unpublished data).

### Molluscicidal performance of the BioBullets

**Figure 3:**
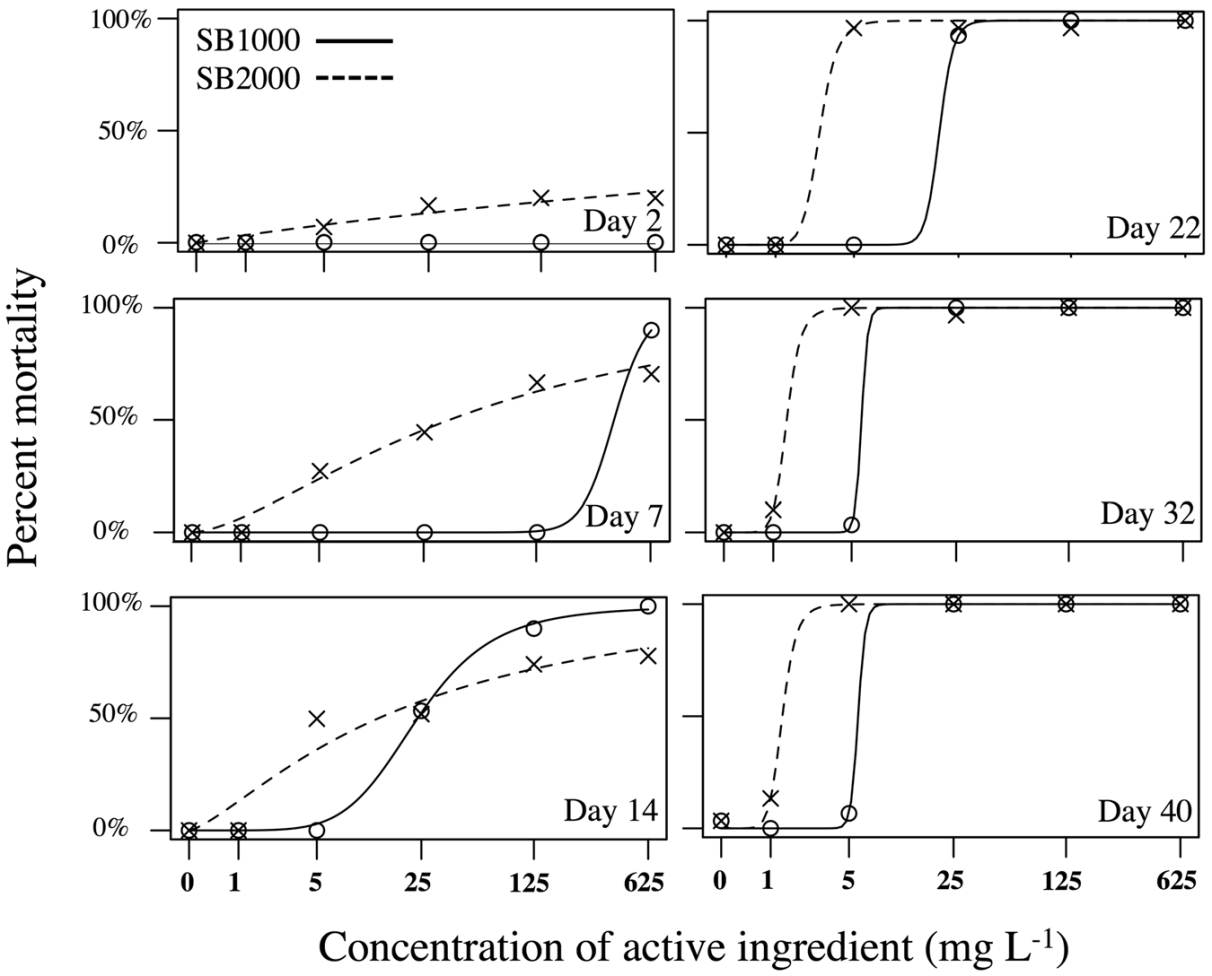
Relationship between percent mortality of *Rangia cuneata* and dose concentration on the 7^th^, 14^th^, 22^nd^, 32^nd^, and 40^th^ days since first exposure of BioBullets product SB 1000 and SB 2000.

Both formulations of BioBullets were highly toxic to *Rangia cuneata*. After 40 days all experimental animals had been killed at concentrations of both products at 25 mg L^−1^ or more (Fig. 3). The mortality in the control group was only one individual (3%), suggesting the high mortality in the treatment groups was associated with the exposure of BioBullets. The mean shell length of *R. cuneata* used in treatment SB1000, SB2000, and control condition were not significantly different (mean = 44.2 ± 9.8 SD mm, F _(2, 302)_ = 0.643, P = 0.526). The shell length of the dead and alive clams at the end of the experiment was similar, indicating no size selectivity (SB1000: F _(1,170)_ = 0.0007, P = 0.979; SB2000: F _(1, 161)_ = 0.427, P = 0.515).

The mortality of *R. cuneata* increased with dose concentration in SB1000 and SB2000 (Fig. 3). During the first two days (48 hours) after exposure, mortality was zero in clams treated with SB1000 and was less than 30% in clams treated with SB2000. Although the dose-response curves were smoother in SB2000 than in SB1000 during the first two weeks after exposure, the mortality observed in SB2000 was equal or higher than in SB1000 at the end of the experiment. More specifically, 100% mortality was achieved with ≥5 mg L^−1^ SB2000 and ≥25 mg L^−1^ SB1000 30 days after the first exposure. After the third week (day 22), the mortality stabilised in the SB2000 treatments but continued to increase in the low concentration (5 mg L^−1^) of SB1000 (Fig. 3). The estimates of LC_50_, _90_ and _95_ are presented in Table 1. The estimates indicated that at least 300 mg L^−1^ of SB1000 or 60 mg L^−1^ of SB2000 is required to achieve 50% mortality within the first week of exposure. However, the concentrations required to achieve LC_50_ or even LC_95_ were well below 10 mg L^−1^ for both products when the clams were exposed to BioBullets for more than 30 days.

**Table 1:** Estimates (±95% confidence intervals) of LC_50_, LC_90_, and LC_95_ for 7, 14, 22, 32 and 40 days after exposure of SB1000 and SB2000 in *Rangia cuneata*.

		SB1000 (mg L^−1^)	SB2000 (mg L^−1^)
Day 7	LC_50_	369.94 ± 11.59	60.61 ± 6.63
	LC_90_	625.45 ± 5.48	—^a^
	LC_95_	755.45 ± 10.79	—^b^
Day 14	LC_50_	23.67 ± 0.53	21.53 ± 2.58
	LC_90_	100.06 ± 5.49	—^a^
	LC_95_	184.84 ± 13.34	—^b^
Day 22	LC_50_	18.87 ± 6.15	2.70 ± 2.12
	LC_90_	23.82 ± 1.29	3.99 ± 1.02
	LC_95_	25.86 ± 0.98	4.60 ± 0.53
Day 32	LC_50_	5.90 ± 1.30	1.42 ± 0.80
	LC_90_	6.60 ± 2.36	2.07 ± 2.03
	LC_95_	6.86 ± 2.77	2.38 ± 2.61
Day 40	LC_50_	5.82 ± 0.64	1.35 ± 0.40
	LC_90_	6.64 ± 1.62	2.00 ± 1.56
	LC_95_	6.95 ± 2.12	2.31 ± 2.37

^a^LC was not estimated as an average of 90% mortality was not achieved even at the highest concentration during this time period.

^b^LC was not estimated as an average of 95% mortality was not achieved even at the highest concentration during this time period.

**Figure 4:**
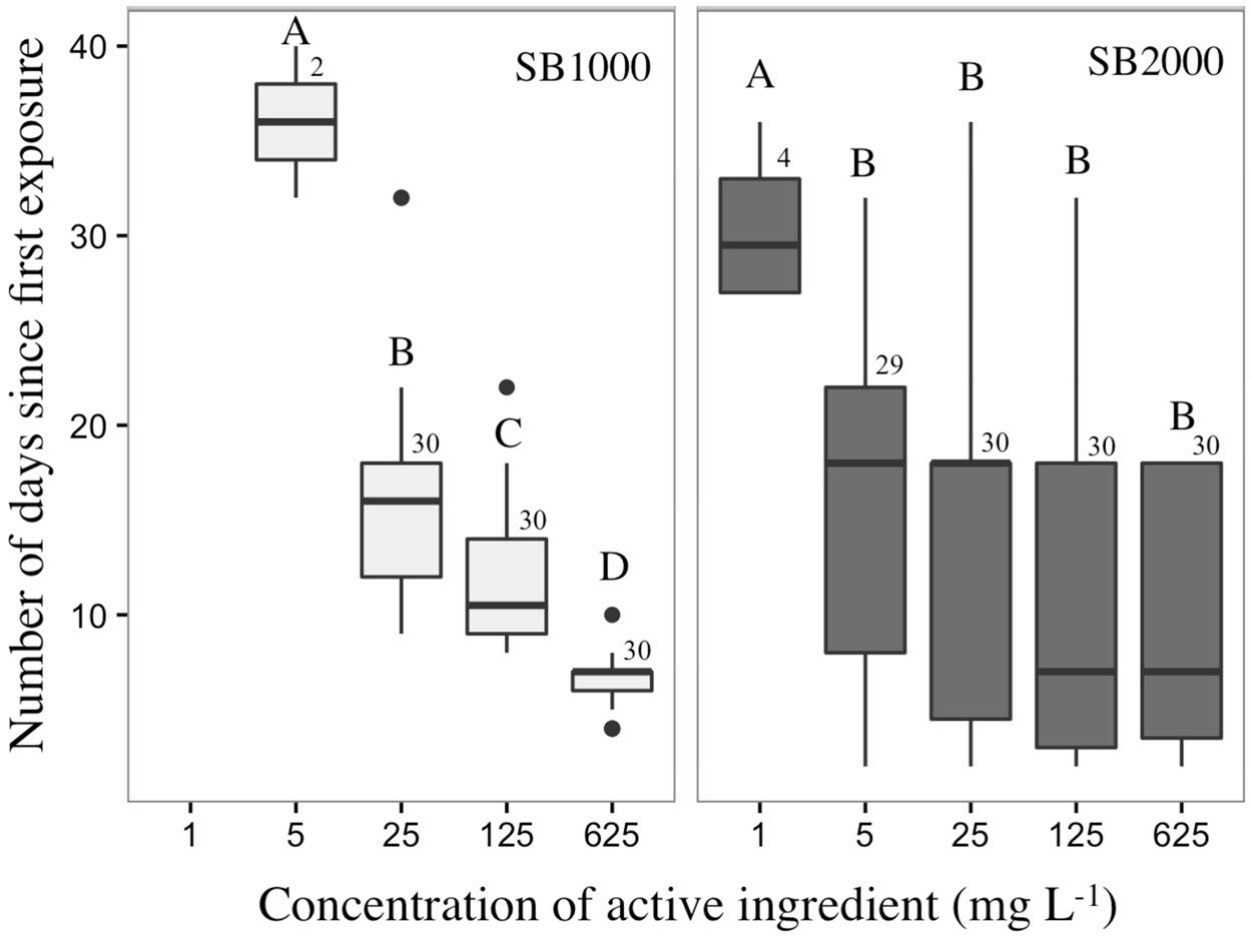
Boxplots of the number of days required to kill *R. cuneata* following the first exposure to SB1000 and SB2000. The horizontal lines inside each box represent the median; lower and upper borders of each box represent the interquartile rage (25th to 75th percentile); whiskers represent the lowest and highest values observed within 1.5 times the interquartile rage; and black dots represent observed values that fall outside this. Numbers above each box represent sample size (n _dead individuals_ in each treatment). Boxes in the same panel that are not connected by the same letter are significantly different (Tukey HSD, α = 0.05).

The number of days required to kill *R. cuneata* following the first and single exposure of BioBullets decreased significantly and negatively with increased concentration of both SB1000 and SB2000 (Fig. 4). The product x concentration interaction was significant, suggesting this dose-dependent response was different for the two products (ANCOVA F _(1, 211)_ = 4.376, P = 0.038). This led us to analyze the dose-dependent response of *R. cuneata* in each treatment type separately. The time required to kill *R. cuneata* gradually decreased with increasing SB1000 concentration (5, 25, 125, and 625 mg L^−1^) (ANOVA F _(3, 88)_ = 51.315, P < 0.0001; all Tukey HSD pairwise comparisons p < 0.05) (Fig. 4). In contrast, the time required to kill *R. cuneata* using SB2000 was only significantly different between 1 mg L^−1^ and rest of the concentration treatments (5–625 mg L^−1^) (ANOVA F _(4, 118)_ = 7.959, P < 0.0001; Tukey HSD p < 0.05 only for 1 mg L^−1^ compared with other treatments) (Fig. 4).

## Discussion

Numerous attempts have been made to combat aquatic pests^19^. Chemical control is the most cost-effective and successful approach^41^. One of the challenges to applying chemical control is to maintain low treatment dosage while preserving the effectiveness of the control agent. By entrapping the biocides into a coating, a filter feeder’s avoidance response is minimised, which allows the accumulation and release of the encapsulant into the filter feeder’s digestive tract. Consequently, compared to using the chemical in its uncoated form, the treatment concentration of encapsulated particles can be lowered substantially, which minimises the impacts on non-target organisms and the wider environment.

With the advanced processing and coating technology used in this study, for the first time, microencapsulated particles were produced in sizes similar to the filter-feeders’ prey. Compared to the previous studies (105.4 ± 59.6 µm^26^ and 101.4 ± 0.1 µm^27^), the particles in the present study were considerably smaller (9.5 ± 0.5 µm SB1000 and 24.6 ± 1.3 µm SB2000). The decrease in the particle size is vital for effectiveness of the performance of biocide, since (1) most filter-feeders capture certain sizes of particles more effectively and these sizes are usually <100 µm, and (2) some filter feeders reject undesirable particles actively even after being captured on the gill^30^. For instance, the preferred prey sizes of two notorious invasive bivalves are both less than 100 µm: <20–25 µm for the Asian clam, *Corbicula fluminea*^42^, and <50 µm for the zebra mussel^43^. Particles that are larger than these sizes are typically rejected and expelled by the animals in the form of pseudofeces^42,43^. By decreasing particle size we may also bring our BioBullets into a size that is readily filtered by non-target species such as zooplankton. In the case of the active ingredient carried within SB1000, the toxicity is especially high to bivalve molluscs but low to other standard test organisms including zooplankton and fish^44^. The active ingredient in SB2000, on the other hand, has similar LC_50_ values for bivalves and zooplankton, but very low toxicity to vertebrates including fish^45^. Therefore, the selection of formulation may be influenced by site-specific conditions, such as the identity of local non-target biota and the level of acceptable collateral damage to recipient communities. In some instances, manufacture of BioBullets of a particular size range could help to reduce uptake by selectively feeding non-target organisms whilst remaining effective against target bivalves.

Reducing the size of microparticles is usually associated with an increase in the rate of release, given that smaller particles have a higher surface to volume ratio than larger ones. Surprisingly, even with the product size decreased substantially, the rate of release did not increase in either of our formulations when compared with previous larger iterations^27^. For the SB1000, although the particle size was decreased five-fold, the rate of release was similar to previous studies, with 90% of the active ingredients released within 3 hours^26,27^. The SB2000 showed a remarkable ability to suppress the release of the encapsulated biocide. Only 50% of the active ingredient was released into the water after 8 hours, and the remaining half of the active ingredient was released into the water in the next 12 hours. The extension of the toxin-releasing process provides a longer period before triggering the avoidance behavior of bivalves, which allows the biocide to accumulate in the animals, and thus lowering the dosage concentration considerably. Designing the optimal release profile is challenging because particles that are too robust will simply pass through the gut without absorption of the active ingredients. Typical transition times through a bivalve from the mouth to the anus is approximately 6–8 hours^46^. Release of active ingredients from ingested particles is facilitated by the digestive action of the animal’s crystalline style.

Interestingly, the surface structure of particle was different between these two formulations. The salt-based active ingredient in the SB2000 appears to have affected the kinetics of crystallisation in a positive way, slowing the release profile. Similar improved release profiles were found in another study when salts were encapsulated within mono- and tri-glycerides^47^, with the salt-driven creation of a sintered fat crystal network that gave total surface coverage with no or very few defects, thus slowing the release profile of the microcapsule. The ‘bird’s-nest’ surface texture of the SB2000 likely reflects the recrystallization of a very small amout of the salt on the capsule surface during cooling. This finding opens up opportunities to modify the surface structure of the product. This could be applied in tailoring species-specific BioBullets in the future, because filter feeding is not simply a sieving process, but a process that is influenced by the surface properties of the particles in addition to the shape and size^43^. Indeed, further specificity might be achieved by targeting surface textures which are accepted by the invasive but rejected by non-target native species.

Our study demonstrated that both of the two new formulations of BioBullets (SB1000 and SB2000), which have been approved for use in UK drinking waters (Regulation 31) and approved for release into open waters, control the newly documented invasive clam, *Rangia cuneata*, effectively even with low dosage. Specifically, a single dose of 30 mg L^−1^ (contains 6 mg L^−1^ active ingredient) of BioBullets SB1000 or a single dose of 10 mg L^−1^ (contains 2 mg L^−1^ active ingredient) of BioBullets SB2000 could achieve 90% mortality after 30 days of the first exposure in a closed environment.

Mortality of *R. cuneata* increased with concentration of BioBullets, which is similar to the previous studies using different formulations of BioBullets to control against zebra mussels^27,28^ and golden mussels^29^. In contrast to the rapid (12–48 hrs) responses to BioBullets when tested against zebra^27,28^ and golden mussels^29^, the response of *R. cuneata* to BioBullets was relatively slow with very low mortality during the first two days of this experiment. This relatively slow response could be due to the different formulations tested and/or the interspecific variation in sensitivity to toxins among these species, or by the relatively cool temperatures (10 °C) used in the present study. Although high mortality was not observed during the beginning of our experiment, the long-term molluscicidal performance of the BioBullets is extraordinary. Importantly, both formulations did not select against body size of *R. cuneata*, indicating their effectiveness across the entire body size range tested. Therefore, our study presents a method of controlling *R. cuneata* using low chemical doses.

The time required to control *R. cuneata* was negatively related to the concentration of BioBullets when using both formulations. However, the dose-response of the *R. cuneata* was different between the two formations. While SB1000 showed a clear gradual decrease of time required to kill *R. cuneata* with the increasing concentrations of BioBullets, this pattern was not found in clams that were treated with SB2000. Instead, when exposed to 5–625 mg L^−1^ of SB2000 BioBullets, most of the mortality occurred during the 10–20 days after the first exposure. In the *R. cuneata* that were exposed to 1 mg L^−1^ of SB2000, the mortality occurred on average 30 days after the first exposure. The contrasting responses of *R. cuneata* to these two formulations could be due to the different physiological effects of the active ingredients or caused by the different physical characteristics in the two formulations. It is also possible that the slower release of the active ingredient of SB2000 BioBullets allowed the accumulation of the lethal concentration in the animals’ disgestive tracts at the beginning of the experiments. The active ingredient in SB2000 then interfered with the cellular respiration of these *R. cuneata* slowly, eventually causing mortality 10 to 20 days after.

Since its first discovery in Belgium in 2005^32^, the Gulf wedge clam, *R. cuneata*, has invaded many other regions of Europe^33–38^, and will continue to spread through Europe. Our study demonstrated a novel approach to control *R. cuneata*. By dosing SB1000 BioBullets at merely 10 mg L^−1^ (containing 2 mg L^−1^ active ingredient), all *R. cuneata* in a closed system can potentially be eliminated.

The quantities of microencapsulated product required to achieve an eradication may preclude its use in large volume, open water settings. However, we estimate that in order to treat the entire 10.4 km drainage channel currently occupied by *R. cuneata* in the UK^36^ would require 4.7 tonnes of SB1000 at a dose of 10 mg L^−1^, which is both economically and logistically feasible. Furthermore, often the aim of a control programme is not to remove every individual of the targeted species from a system, but to reduce its population density below a sustainable level^48^. A density threshold of a control programme can be set either at a density where population growth is negative^49,50^, or at a density that the maximum conservation impacts caused by the pest are acceptable^51^. Reducing the density of invasive species in a system will not only decrease the ecological and economic impacts to the invaded environment but also reduces the possibility that a species can be transported to other locations. Therefore, the BioBullet concentration required to control an invasive bivalve could be lowered if the goal is to reduce the population density rather than eradicate all individuals. It should also be noted that the mortality of *R. cuneata* was only monitored for 40 days during this experiment. It is plausible that the mortality may be higher over a longer timeframe than was used in our laboratory tests. Therefore, mortality of invasive bivalves in an open water system may ultimately be higher than that observed in the laboratory when treated with the same concentration of BioBullets.

Compared to conventional chemical treatments, microencapsulation of control agents offers a more environmentally-friendly approach to management of invasive bivalves, as it offers a targeted solution that reduces impacts on native non-target biota. Beyond the potential of using BioBullets to control *R. cuneata*, the advanced technology of encapsulating biocide may be used in managing other invasive suspension feeders such as sponges, bryozoans, hydrozoans, as well as some crustaceans. By modifying the active ingredient, the coating material, the surface structure and particle size, species-specific BioBullets could be tailored for each invasive species.

As the spread of aquatic invasive species continues and novel control options emerge, attempts to eradicate or reduce populations should be considered more often. Tried-and-tested eradication and containment tools, such as BioBullets, offer the opportunity for the rapid response to newly detected aquatic invasive non-native species and help to support an effective risk management plan.

## Material and Methods

### Formulation and manufacture of the microencapsulated BioBullets

We manufactured lipid-walled microparticles containing one of two active ingredients under patent no. GB0001281D0 to BioBullets Ltd. (Cambridge, UK). These two new formulations have been approvided by the Drinking Water Inspectorate for use in UK drinking waters. In addition, independent assessment by regulatory authorities concluded that sufficient evidence exists to support the assumption that, following breakdown of BioBullets, the active ingredients of both SB1000 and SB2000 will sorb to organic material in water, such that aquatic phase concentrations should be negligible. The active ingredient in SB1000 is a cationic polymer that functions as a surfactant. Surfactants are toxic to bivalves because they adsorb at the plasma membrane, including gill tissues, disrupting transfer mechanisms between the cells and the surrounding medium and causing cytolosis^44^. The active ingredient in SB2000 is an organic, anionic salt known to interfere with mitochondrial metabolism and cellular respiration. Salts can be especially toxic to bivalves because they typically have dilute body fluids and are therefore susceptible to osmotic shock^52^.

A premix slurry was prepared containing the encapsulant and active ingredient under conditions of controlled shear. The slurry was pumped into an ultrasonic atomising nozzle at the top of a cooling chamber. The atomised particles formed perfect spheres as they cooled and fell to the chamber base. Further particle cooling was achieved with an air-conveyancing system before discharge via cyclone to a fluid bed processor. The encapsulated particles were then coated with non-ionic surfactant to aid dispersion in water. Further cooling in the fluid bed removed all heat of crystallisation from the microparticles before packaging. Both products carried 20% active ingredient by weight.

These new formulations of BioBullets represented substantial improvements on those previously reported^26,27^. Smaller sizes of particles was achieved through three key innovations: (i) faster peripheral velocity using a rotary atomiser yielded smaller droplets; (ii) refined machining, surface finish and profiling of the atomisation disc imparted more energy into the liquid film which resulted in improved atomisation; (iii) improved rheological properties of the premix slurry created a lower viscosity premix which thus generated smaller sized microcapsules.

### Physical characteristics of the BioBullets

The microstructure of the microencapsulated particles was visualized through scanning electron microscopy (Quanta 650 F, 2 kV, spot size = 1.5 μm). Approximately 50 mg of uncoated particles were mounted in a SEM sample stub using double-sided carbon tape. To determine the size distribution of the encapsulated particles, the BioBullets samples were dispersed in ultra-pure water, and the size of each particle was determined by a Coulter LS230 light scattering device.

The ability of the encapsulation process to suppress the release of the active ingredient was analysed by conductivity measurements. For each of the formulations, 1 g particles were placed in a 1-L beaker containing 500 mL of 25 °C deionised water. To prevent water losses by evaporation, the beaker was covered with cling film. To ensure particles were suspended in the aqueous medium, a magnetic stirrer was used to stir the mixture at approximately 60 rpm. The conductivity of the aqueous medium was monitored for 24 hours using a conductivity meter (Jenway, Model 4310) and conductivity cell. The conductivity of water was recorded every one minute for the first 4 hours for SB1000 and the first 2 hours for SB2000, then every 15 minutes for the next 20 hours. The measured conductivities were then converted into active ingredient concentration values using a calibration curve. The percent of the active ingredient released from the encapsulated particles was calculated as the active ingredient concentration measured at a given time divided by the active ingredient concentration measured at the end of the tests.

### Molluscicidal performance of the BioBullets

*Rangia cuneata* were collected from the South Forty-Foot Drain (N52.967147, W -0.028836) by the Environment Agency on December 9^th^, 2016. Specimens were collected randomly by hand net (mesh size 0.5 mm) to ensure a representative sample of the entire clam population was tested. The shell length of *R. cuneata* ranged from 18 mm to 64 mm. Immediately after collection, the *R. cuneata* were transported to the laboratory at the University of Cambridge in field water. This study was conducted in a constant temperature controlled room at 10 °C (±1 °C), which matches the water temperate recorded on the day of collection. The clams were acclimated for 8 hours prior to exposure.

Given the low flow in the South Forty-Foot Drain, the laboratory tests were conducted using static conditions rather than flow-through. To test the performance of the BioBullets, ten individual clams were placed randomly into each of 33 3-L containers with 2 L of aerated water from the collection site (~1.4‰). The clearance rate of the ten individuals in each container was ~ 3 L h^−1^ estimated based on the average biomass (8.9 g) in each container clearance rate (0.34 L g^−1^ hr^−1^) of *R. cuneata* reported in a previous study^53^. This suggested that the individuals in each container are capable of filtering the entire volume of treatment water within hours. In addition, river water from the collection sites was used throughout the experiments, which enabled the exposed active ingredient of both SB1000 and SB2000 to sorb to organic material in water and thus become rapidly inactivated.

*R. cuneata* were exposed to a single dose of 5, 25, 125, 625, 3125 mg L^−1^ of either SB1000 or SB2000 which contained 1, 5, 25, 125, 625 mg L^−1^ of active ingredient. Control organisms were held in identical conditions but not exposed to toxins. Each of the 11 treatments was tested in triplicate. The replications were randomly distributed within the temperature-controlled room. Mortality was inspected daily during the first 12 days of the experiment, then on a regular basis until the experiment was terminated at day 40. Monitoring of clams was conducted over 40 days because we anticpated a delay between exposure to the toxicants and mortality. In total, 18 inspections were made over the course of the experiment. Mortality was identified by the lack of valve closure in response to a blunt probe. Dead organisms were removed immediately to avoid contamination of the holding water. Shell length of *R. cuneata* was measured (±1 mm) with calipers

### Statistical analyses

Size structure of the encapsulated particles for each formulation was constructed by separating particles with diameter <1 µm into 0.1 µm size bins, and then all the particles with diameter ≥1.00 µm were separated into 1 µm size bins. The differential volume (%) of each size bin was then calculated as the number of particles in each bin divided by the total number of particles in each formulation. The biocide dissolution kinetics was expressed as the percent of the active ingredient released from the encapsulated particles to the aqueous medium over 1,440 minutes (24 hours).

Mortality was calculated based on the number of organisms that died during the defined period. Dose–response curves were generated using a two-parameter logistic model using the package “drc” within the R version 3.5.1 (R Development Core Team, Vienna, Austria)^54^. Lethal concentration (LC) 50, 90, and 95 and their 95% confidence intervals (95% CI) was predicted for the 2^nd^, 7^th^, 14^th^, 22^nd^, 32^nd^ and 40^th^ days after exposure. We also used ANCOVA to test the influence of product type (SB1000 and SB2000) and concentration of the product on the time (days) required to kill organisms since the first exposure. Organisms that were not killed were excluded in this analysis. To assess the selectivity of BioBullets on the body size (shell length), ANOVAs were preformed to compare the shell length of dead/alive organism in SB1000 and SB2000 separately. ANCOVA and ANOVA analyses were performed using R^54^. All concentration values quoted are for active ingredient (AI).

### Ethics

All applicable international, national, and/or institutional guidelines for the care and use of animals were followed.

## Data Availability

Data are available at: https://mfr.osf.io/render?url=https://osf.io/yh68k/?action=download%26mode=render.
